# DNA vaccine VGX-3100 with electroporation induces regression of cervical intraepithelial neoplasia 2/3 and clears HPV infection with robust T cell responses: results of a randomized, double-blind, placebo-controlled Phase II trial

**DOI:** 10.1186/2051-1426-2-S3-O17

**Published:** 2014-11-06

**Authors:** Laurent Humeau, Cornelia Trimble, Matthew Morrow, Xuefei Shen, Michael Dallas, David Weiner, Jean Boyer, Jian Yan, Kimberly Kraynyak, Albert Sylvester, Mary Giffear, Kathy Marcozzi-Pierce, Divya Shah, Kate Broderick, Amir Khan, Jessica Lee, Niranjan Sardesai, Mark Bargarazzi, the HPV-003 Protocol team

**Affiliations:** 1 Inovio Pharmaceuticals, USA; 2 Johns Hopkins, Baltimore MD, USA; 3 Perelman School of Medicine University of Pennsylvania, PA, USA

## 

The Phase II study, designated HPV-003 (NCT01304524), assessed the safety and efficacy of VGX-3100 in 167 women with biopsy-proven cervical intraepithelial neoplasia (CIN) 2 or CIN 3 and HPV-16 or -18. The randomized, placebo-controlled, double-blind study, was stratified by age and severity of CIN and evaluated cervical tissue changes after three 6 mg intramuscular doses of the DNA vaccine VGX-3100 followed by electroporation (EP) with Inovio's CELLECTRA^®^ 2000 device at weeks 0, 4, and 12. Cervical tissue was examined before starting blinded treatment and ~9 months later. The primary endpoint was regression of CIN 2 or CIN 3 to CIN 1 or no disease at 6 months post third dose. In the per-protocol population (PPP), lesions regressed in 53 of the 107 women receiving VGX-3100 (49.5%) as compared to 11 of the 36 women in the placebo group (p < 0.025). The secondary endpoint was to demonstrate HPV-16 or -18 clearance from the cervix in conjunction with regression of CIN 2/3 to CIN1 or no disease. Among the 107 women in the VGX-3100 group, 43 demonstrated regression and virological clearance (40.2%), compared to 5 out of 35 (14.3%) in the placebo group (p < 0.025).

The study also explored cell mediated immune responses to VGX-3100 in blood samples taken prior to the first vaccine dose and periodically thereafter. IFN-γ ELISpot results revealed higher responses in the VGX-3100 treated group than in the placebo group. Flow cytometry and immunohistochemistry analyses are also ongoing. Finally, subjects were also monitored for tolerability and safety. The treatment was generally well-tolerated, with only administration site redness occurring significantly more frequently in the VGX-3100 group compared to the placebo group in the 7- and 28-day periods following treatment.

Altogether, the successful Phase II results clearly illustrate the highly promising potential of therapeutic vaccination with DNA followed by electroporation for the treatment of HPV-related precancerous cervical disease in women as well as HPV-associated cervical, head and neck, and anogenital cancers.

